# Modeling Activity and Target-Dependent Developmental Cell Death of Mouse Retinal Ganglion Cells *Ex Vivo*

**DOI:** 10.1371/journal.pone.0031105

**Published:** 2012-02-17

**Authors:** Sylvie Voyatzis, Aude Muzerelle, Patricia Gaspar, Xavier Nicol

**Affiliations:** 1 Institut National de la Santé et de la Recherche Médicale, Unité mixte de Recherche en Santé 839, Paris, France; 2 Université Pierre et Marie Curie, Paris, France; 3 Institut du Fer à Moulin, Paris, France; National Institutes of Health/NICHD, United States of America

## Abstract

Programmed cell death is widespread during the development of the central nervous system and serves multiple purposes including the establishment of neural connections. In the mouse retina a substantial reduction of retinal ganglion cells (RGCs) occurs during the first postnatal week, coinciding with the formation of retinotopic maps in the superior colliculus (SC). We previously established a retino-collicular culture preparation which recapitulates the progressive topographic ordering of RGC projections during early post-natal life. Here, we questioned whether this model could also be suitable to examine the mechanisms underlying developmental cell death of RGCs. Brn3a was used as a marker of the RGCs. A developmental decline in the number of Brn3a-immunolabelled neurons was found in the retinal explant with a timing that paralleled that observed *in vivo*. In contrast, the density of photoreceptors or of starburst amacrine cells increased, mimicking the evolution of these cell populations *in vivo*. Blockade of neural activity with tetrodotoxin increased the number of surviving Brn3a-labelled neurons in the retinal explant, as did the increase in target availability when one retinal explant was confronted with 2 or 4 collicular slices. Thus, this ex *vivo* model reproduces the developmental reduction of RGCs and recapitulates its regulation by neural activity and target availability. It therefore offers a simple way to analyze developmental cell death in this classic system. Using this model, we show that ephrin-A signaling does not participate to the regulation of the Brn3a population size in the retina, indicating that eprhin-A-mediated elimination of exuberant projections does not involve developmental cell death.

## Introduction

During the development of the central nervous system, neurogenesis and programmed cell death occur concomitantly. Developmental cell death plays different roles in morphogenesis: regulation of the size of progenitor population in the CNS, removal of damaged cells, optimization of cell population matching between interconnected neurons and removal of neurons with ectopic connections [1]. In the mouse retina, the main wave of histogenetic cell death occurs during the first two postnatal weeks, coinciding with the formation of retinotopic maps in the superior colliculus (SC). All retinal cell types undergo developmental cell death, with different time-courses [2], [3] but retinal ganglion cells (RGCs) are the ones undergoing substantial developmental cell death. While 5% of the photoreceptors undergo developmental cell death, up to 50% of RGCs are lost due to cell death, peaking between P2 and P4 in mice [3], [4]. However, the methods used to quantify the reduction in RGC number have often been indirect, and have led to a large variability in the results, ranging from 9% [3] to 90% [5].

The size of the RGC population is dependent on electrical activity [6] and is regulated by competition for trophic factors between retinal axons in their targets [7]–[10]. The reduction of RGC number participates in the elimination of subpopulations of ectopically projecting RGCs [11], [12]. In the adult, RGC axons are topographically organized in their targets, with the temporo-nasal axis of the retina projecting on the rostro-caudal axis of the SC [13], [14]. Temporal axons first overshoot their final arborization zone in the rostral part of the SC, reaching the caudal SC. Topography is subsequently refined during the first post-natal week in mice by eliminating exuberant projections [15]–[17]. Blocking electrical activity in the retina promotes the survival of the ectopically projecting temporal RGCs [18], [19]. Similar mechanisms were found to operate for the establishment of bilateral retinal projections: most of the RGCs project contralaterally [20] and lack of neural activity maintains RGCs projecting to the ipsilateral SC that are normally eliminated [11]. However, it remains unclear whether developmental cell death has a role in the elimination of all populations of ectopically projecting RGCs.

Specific molecular markers that are expressed in a large population of RGCs are now available. Such markers allow a direct estimation of RGC number. For instance the POU domain transcription factor Brn3a labels a large population of RGCs. The Brn3a RGCs were shown to contribute only to the principal retino-thalamic and retino-collicular visual pathways and to be excluded from the subcortical pathways of the accessory optic system, moreover they project mainly contralaterally [21]. We used this marker to evaluate the number of RGCs in the retina at several time-points during the first postnatal week in mice. We analyzed a previously established *ex vivo* retino-collicular co-culture mimicking the development of neuronal network between the retina and the SC [22]. We show that the time course of the reduction of RGC number is similar to that occurring *in vivo*. In this co-culture, electrical activity regulates the size of the RGC population, mimicking *in vivo* observations. Similarly, manipulation of the target size enabled an evaluation of the influence of competition for space between retinal axons on RGC survival. We used this model to assess the influence of ephrin-As and the topographic organization of the retino-collicular projections on the population size of a genetically defined population of RGCs.

## Results

### Reduction of the number of Brn3a-expressing RGCs between P0 and P7

**Figure 1:**
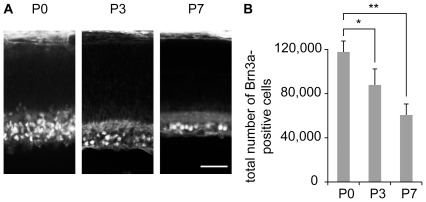
The number of Brn3a-expressing RGCs undergoes a reduction during the first postnatal week. (A) P0, P3, and P7 retinal sections were immunostained with a Brn3a antibody. The inner surface of the retina (RGC layer) is down. The density of Brn3a-expressing cells decreases from P0 to P7 and the retinal ganglion cell layer reorganizes from a multiple cell layer at P0 to a single cell layer at P7. (B) The total number of Brn3a-positive RGCs in the retina declines between P0 and P7. n≥3 for each age. Error bar, s.e.m.; scale bar 50 µm; * p<0.05, ** p<0.01, Kruskall-Wallis test.

Brn3a expression is limited to post-migratory RGCs, beginning at E12.5 with a stable expression until adulthood [21]. To estimate changes in Brn3a-expressing RGC number during the period of developmental cell death, we immunostained serial sections of P0, P3 and P7 retinas for Brn3a, and estimated the total number of Brn3a RGCs in the retina. Between P0 and P7, the population of Brn3a-positive cells in the retina decreased by ∼48% (Fig. 1A and 1B). Concurrently with the decline of Brn3a-expressing RGC, retinal surface increased, and, the RGC layer of the retina reorganized into a single cell layer (Fig. 1A).

**Figure 2:**
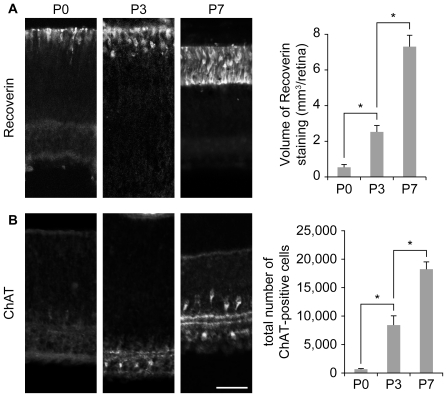
The number of photoreceptors and starburst amacrine cells increases during the first postnatal week. (A) Recoverin and (B) ChAT immunoreactivity reveals photoreceptors and starburst amacrine cells respectively. Both cell types undergo an expansion of their population between P0 and P7. n≥3 for each age. Error bar, s.e.m.; scale bar 50 µm; * p<0.05, Kruskall-Wallis test.

To evaluate whether reduction in cell number was a general feature of all retinal cell types during the first post-natal week, retinal sections were stained for recoverin, a marker for photoreceptors [23], or choline acetyltransferase (ChAT), a marker for starburst amacrine cells [24], [25], a subpopulation of interneurons in the inner nuclear layer. Between P0 and P7 both recoverin- and ChAT-positive cell populations expanded (Fig. 2A and 2B). Thus, the reduction in the number of Brn3a-positive RGCs during the first postnatal week does not reflect a general loss of all retinal cell types. The observed timing of reduction of Brn3a-positive RGCs coincides with the period of maximal developmental cell death of RGCs described in rodents [3] and falls within the range of previous numerical estimates (9 to 90% [3], [5], [26]–[28]).

### Reduction of RGC number is mimicked in a retino-collicular co-culture

**Figure 3:**
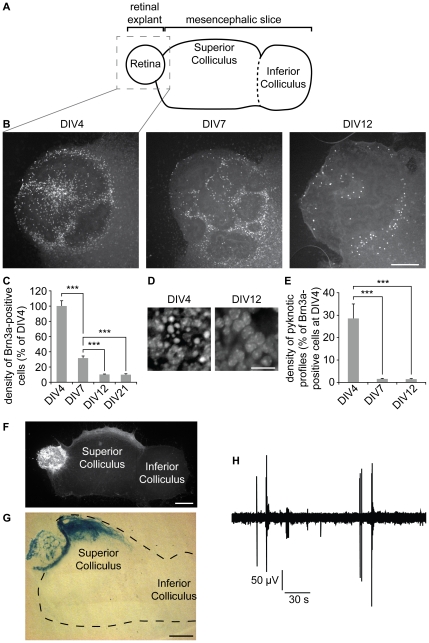
The number of Brn3a-expressing cells in retino-collicular co-cultures decreases between DIV4 and DIV12. (A) Schematic of the retino-collicular co-culture. A retinal explant is placed in contact with the rostral end of a parasagittal mesencephalic slice containing the SC. (B, C) The density of cells immunoreactive for Brn3a undergoes a reduction of ∼68% between DIV4 and DIV7, and a further decrease of ∼22% between DIV 7 and DIV12. No further reduction is observed between DIV12 and DIV21. The density measured for each age was normalized to the density of Brn3a-positive cells at 4DIV. (D, E) Density of dying cells revealed by the number of bisbenzimide-stained pyknotic profiles, and measured at DIV4, DIV7 and DIV12. The density of pyknotic profiles was normalized to the density of Brn3a-positive cells at DIV4 to coarsely estimate the percentage RGCs lost at each age. This estimation would only give an order of magnitude of the percentage of dying Brn3a-positive RGCs because only ∼30% of RGCs express Brn3a and other cell types are present in the retinal explant. (F) Axons from GFP expressing retinal explants invade the superficial layers of the SC at DIV12. (G) Projections from Brn3a-positive RGCs are detected in the SC using a retinal explant from a Brn3a-LacZ reporter mouse line. (H) Field electrophysiogical recording of the collicular slice at DIV12. Large bursts of spontaneous correlated activity are detected. B, scale bar 200 µm. D, scale bar 50 µm. F,G, scale bar 500 µm. Error bar, s.e.m.; n≥10 cultures per condition; *** p<0.001, ANOVA.

We examined the maturation of these retinal cell types in an *ex vivo* model which combines an E15.5 retinal explant and a parasagittal slice of SC, one of the targets of Brn3a RGCs (Fig. 3A) [22]. The preparation was fixed, immunostained for Brn3a and counterstained with bisbenzimide to identify the pyknotic profiles of dying cells after 4, 7, 12 or 21 days *in vitro* (equivalent to P0, P3, P7 and P12 respectively for the age of the retinal explant). We observed a ∼68% decrease in the density of the Brn3a positive neurons from DIV4 to DIV7, and a further ∼22% decrease in the density from DIV7 to DIV12 (Fig. 3B and 3C). The initial reduction of Brn3a-positive RGCs coincided with a high number of pyknotic profiles (Fig. 3D and 3E), indicating that a large amount of cells are undergoing cell death. No significant change in RGC number was observed between DIV12 to DIV21 indicating a long term survival of RGCs after the initial massive loss (Fig. 3C). There was also a gradual histotypic organization of the Brn3a-positive cells that became organized in a layer generally at the periphery of the retinal explant and to a lesser extent in the center of the explant (Fig. 3B). This elimination of Brn3a-positive cells is therefore similar to the reorganization of RGCs *in vivo* between P0 and P7 that leads to the formation of a single cell layer (Fig. 1A). Retinal explants from mouse reporter lines expressing GFP under the control of the β-actin promoter [29] or β-galactosidase under the control of the Brn3a promoter [30] were used to verify that RGC axons were invading the superior colliculus *ex vivo*. In both conditions, GFP-positive or LacZ-positive axons were found in the collicular slice at DIV12, invading specifically the superficial layers of the SC (Figure 3F and 3G) as previously described [19], [22]. To ensure that the collicular slice was still alive at the end of the *in vitro* period, extracellular electrophysiological recordings of the mesencephalic slice were performed. Large bursts of spontaneous correlated activity were detected, confirming the viability of the collicular slice during the entire culture period (Figure 3H).

**Figure 4:**
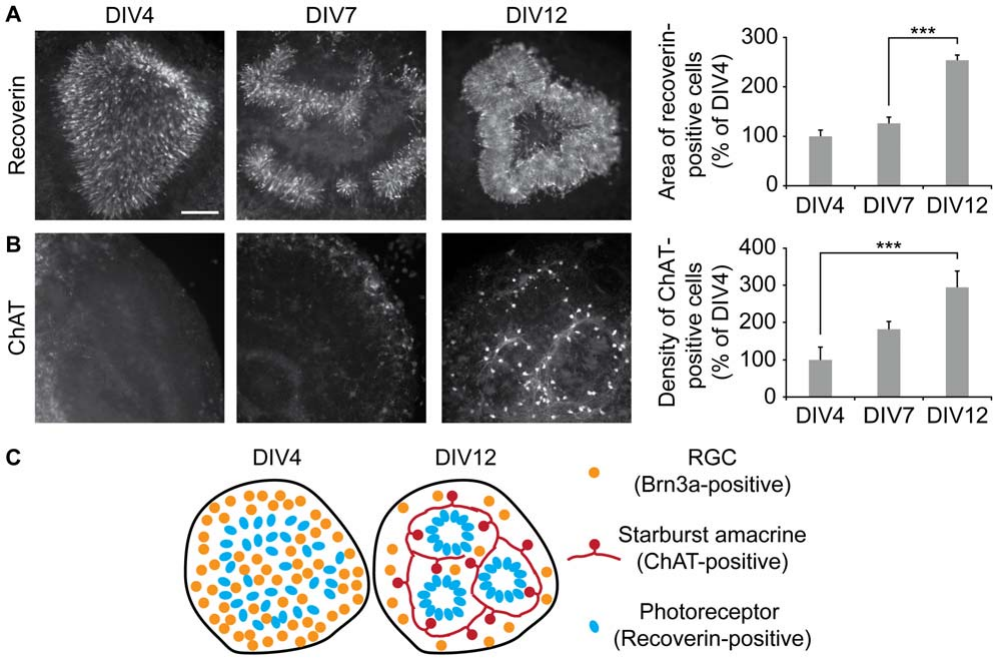
Photoreceptor and starburst amacrine cell populations expand in the retino-collicular co-cultures between DIV4 and DIV12. (A) Recoverin and (B) ChAT immunoreactivity reveals photoreceptors and starburst amacrine cells respectively in the retinal explant of retino-collicular co-cultures. Both cell types undergo an expansion between DIV4 and DIV12. Scale bar 100 µm; Error bar, s.e.m.; n≥7 cultures per condition; *** p<0.001, ANOVA. (C) Reorganization of the retinal explant *ex vivo*. At DIV4, RGCs (orange) and photoreceptors (blue) are mixed in the explant. After 12 days *in vitro*, RGCs are preferentially located at the border of the retinal explant, while photoreceptors form rosette-like structures in the center of the explant. ChAT amacrine cells (red) are positioned between RGCs and photoreceptors.

We evaluated the evolution of photoreceptors and starburst amacrine cells in the retino-collicular *ex vivo* model, by immunostaining the cultures for recoverin or ChAT respectively. The density of photoreceptors increased between DIV4 and DIV12, and their organization in the retinal explant switched from a uniform distribution to enrichment within the center of the explants (Fig. 4A). Few starburst amacrine cells were detected at DIV4 and their number increased progressively at DIV7 and DIV12. At DIV12, starburst amacrines were located between the RGCs and the photoreceptors, a position similar to that observed *in vivo* (Fig. 4B). The expansion of the recoverin- and ChAT-positive population size shows that there is ongoing cell division in the retinal explant.

Overall, this analysis showed that the development of the retinal explant in the retino-collicular co-culture system follows the histotypic reorganization of the retina *in vivo*, and mimics the reduction of RGC number that occurs during the first post-natal week (Fig. 4C).

### Reduction in RGC number is activity-dependent

**Figure 5:**
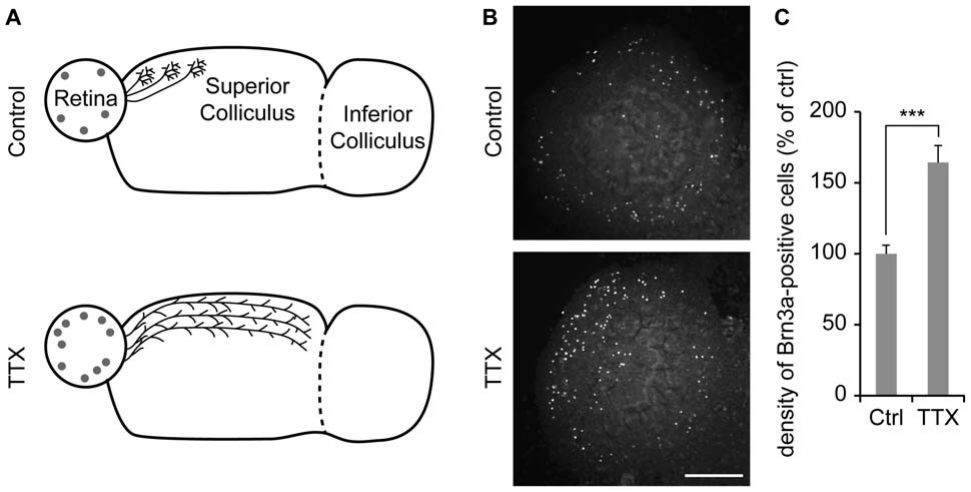
Blockade of electrical activity limits the reduction in the number of Brn3a-expressing cells. (A) TTX treatment prevents the retinotopic organization of axonal arbors *ex vivo*. Temporal axons arborize in the rostral SC in control conditions. In contrast, electrical activity blockade with TTX abolishes the preference of axons from the temporal retina for the anterior SC as described in [19]. Grey circles symbolize Brn3a-positive RGCs and their density is representative of the number of Brn3a-labeled cells in each condition. (B) Incubation of retino-collicular co-cultures in the sodium channel blocker TTX increases the number of Brn3a-immunoreactive cells in retinal explants at DIV12. (C) TTX treatment causes a ∼64% increase in the number of Brn3a-expressing cells at DIV12. Error bar, s.e.m.; Scale bar 200 µm; n≥22 cultures per condition; *** p<0.001, ANOVA.

One of the proposed functions for RGC selective death is to complement the topographic refinement of the retinotopic map that is initially coarsely organized in the rodent SC, particularly across the rostro-caudal dimension [16]. The refinement of retino-collicular maps is dependent on electrical activity of RGCs [31] and can be partially explained by the activity-dependent preferential cell death of RGCs that overshoot their termination zone [11], [12]. However, the influence of electrical activity on the total number of RGCs is largely unknown. We have previously demonstrated that the rostro-caudal refinement of the topography is reproduced in the retino-collicular slice preparation [22] and that the sodium channel blocker tetrodotoxin (TTX) causes retinal axons to maintain exuberant axons in inappropriate targets [19] (Fig. 5A). We evaluated the regulation of Brn3a-positive RGC number, in retino-collicular co-cultures in which TTX was used to block neuronal activity during the entire culture period. TTX treatment induced a ∼64% increase in the number of Brn3a-positive cells at DIV12 compared to control cultures (Fig. 5B and 5C). We conclude that neural activity contributes to the elimination of Brn3a-positive RGCs.

### RGC number reduction is dependent on target availability

**Figure 6:**
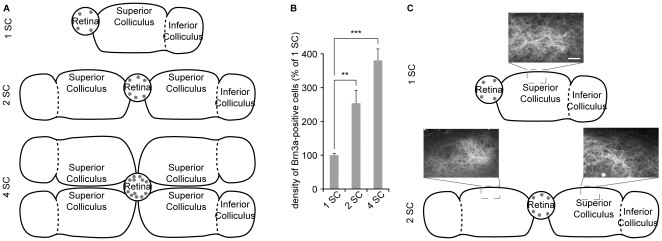
Increasing the size of the target reduces the elimination of Brn3a-expressing cells. (A) The modulation of target availability was achieved by increasing the number of mesencephalic slices in contact with the retinal explant. Grey circles symbolize Brn3a-positive RGCs and their density is representative of the number of Brn3a-labeled cells in each condition. (B) The density of Brn3a-immunoreactive cells in retinal explants confronted with 2 (2 SC) or 4 (4 SC) collicular slices was increased by ∼153% and ∼280% respectively at DIV12 in comparison with parallel cultures with a single collicular slice. (C) Axonal arbors from GFP-expressing retinas are detected in the superior colliculus of all mesencephalic slices that are available to them. Error bar, s.e.m.; Scale bar 50 µm; n≥10 cultures per condition; ** p<0.01, *** p<0.001, ANOVA.

Competition for target availability and neurotrophic factors regulates survival of RGCs as shown in monocular deprivation or partial collicular ablation experiments [7]–[11], [32], However testing this hypothesis by increasing the size of the target has not been possible *in vivo*. The retino-collicular co-culture model enabled the manipulation of the target size, by varying the number of collicular slices in contact with the retinal explant (Fig. 6A). Doubling the target size reduced the loss of Brn3a-positive cells at DIV12 and multiplying the target size by 4 led to a further reduction of cell death (Fig. 6B). Increasing the number of mesencephalic slices that faced a single retinal explant did not modify the pattern of retinal projections in each superior colliculus (Fig. 6C). This suggests that retinal axons compete for space in the SC, and that one of the results of this competition is the elimination of Brn3a-positive RGCs.

### Ephrin-A-induced retinocollicular topography does not influence RGC number reduction

**Figure 7:**
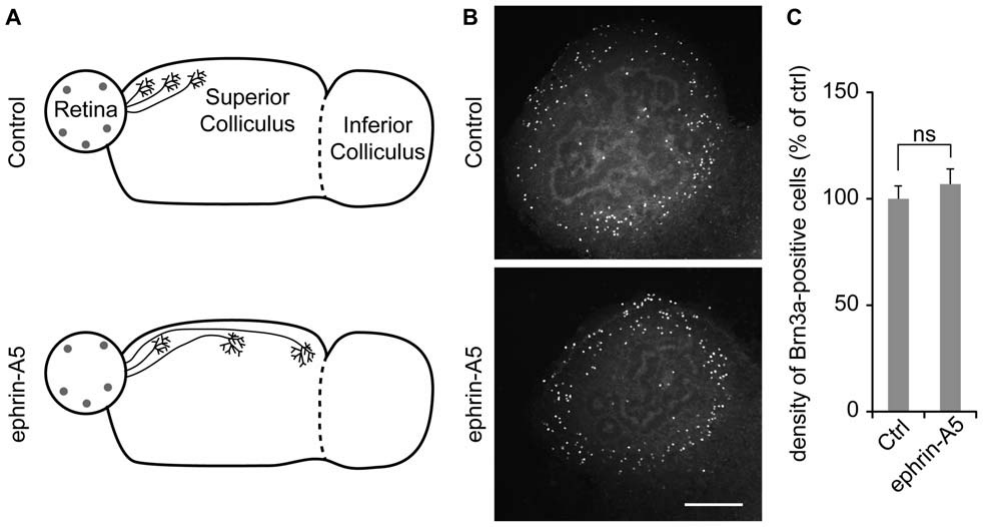
Blockade of ephrin-A signaling does not affect Brn3a-expressing cell number reduction. (A) Ephrin-A5 treatment prevents the retinotopic organization of axonal arbors *ex vivo*, as described in [22]. Temporal axons arborize in the rostral SC in control conditions. In contrast, overall activation of ephrin-A/EphA signaling with ephrin-A5 abolishes the preference of axons from the temporal retina for the anterior SC. Grey circles symbolize Brn3a-positive RGCs and their density is representative of the number of Brn3a-labeled cells in each condition. (B, C) The density of Brn3a-immunoreactive cells in retinal explants is not affected by application of ephrin-A5 between DIV1 and DIV12 (0.5 µg/µl). Error bar, s.e.m.; Scale bar 200 µm; n≥40 cultures per condition; ns, not significant, ANOVA.

In addition to being regulated by electrical activity and competition for target availability, connectivity between the retina and its target is modulated by ephrin-A-dependent axonal repulsion [33]. Topography of retinal projections is dependent on ephrin-As *in vivo* [33]. Ephrin-A signaling regulates apoptosis of neuronal progenitors in the cerebral cortex [34], but its involvement in the regulation of the RGC population size is unclear. To evaluate the involvement of ephrin-As in the regulation of RGC number, we activated all EphA receptors in the retino-collicular co-cultures with ephrin-A5 incubation from DIV1 to DIV12. We have previously showed that this treatment prevented the development of the topography in the *ex vivo* retino-collicular system [22] (Fig. 7A). In contrast however, ephrin-A5 treatment did not affect the number of Brn3a-positive cells in the retinal explant at DIV12 (Fig. 7B and 7C), demonstrating that ephrin-A signaling does not influence the number of Brn3a-expressing RGCs, and suggesting that topographic errors do not influence the elimination of RGCs.

## Discussion

### Direct estimation of RGC loss during the first postnatal week

We monitored the development of Brn3a-expressing RGCs during the first postnatal week. Brn3a is a marker of a subpopulation of RGCs with specific projections to the thalamus and colliculus [21]. Our direct measurements *in vivo* approximate the amount of RGC loss between P0 and P7 to ∼48%, which falls within the large range of previous approximations and refines the previous estimations performed using indirect methods [3], [5], [26]–[28]. This included the quantification of pyknotic profiles in the inner nuclear layer (RGC layer) [3] which can overestimate cell death as it does not allow to distinguish RGCs from displaced amacrine cells, that account for 50% of the cells in the RGC layer [35], [36]. In contrast, retrograde labeling using tracer injections [27] is highly dependent on the size of the injection and may underestimate cell death. The comparison of the number of axons in the optic nerve with the number of cells in the RGC layer [28] is also affected by the presence of amacrine cells that are present in the RGC layer. This method also assumes that all RGC axons have a single branch in the optic nerve.

### A new model to study developmental reduction of RGC population size

The evaluation of factors influencing developmental cell death *in vivo* requires the manipulation of the retina, which is usually done with injection of pharmacological agents in the eye or in the retinal targets in the brain [18]. Both manipulations are not possible before birth in mammals and recurring intravitreal injections are challenging in young pups of mice without damaging the retina and potentially affecting RGC survival, although this technique has been previously used in larger rodents like rat [8]. We describe here the use of an *ex vivo* model for the study of the developmental regulation of RGC population size, that enables pharmacological manipulations, including early developmental stages corresponding to embryonic ages. In addition, transgenic animals with lethal mutations at late embryonic stages or after birth can also be used [19], since the retina is dissected out at embryonic stages. Furthermore, it is possible to mix different genotypes of transgenic mice for the retina and its targets offering a convenient way to evaluate the respective roles of molecules in the retina or in the target cells [22]. This experimental model has been previously used to assess the development of retino-collicular topography and mimics the development of retinal projections and its dependence on ephrin-A signaling [22]. The development of retinal electrical activity follows *ex vivo* a similar time course as its maturation *in vivo* [19]. Here, we extended the repertoire of events showing close similarities in the maturation of the retina *in vivo* and *ex vivo*. Thus, the development of photoreceptors, starburst amacrine cells and RGCs follows a similar time course *in vivo* and *ex vivo*, although there is a noticeable acceleration of RGC cell death at the beginning of the culture period as previously reported [37] and which may be related to the axotomy of retinal axons that had already reached their brain target at the time when the retinal explants are dissected. Interestingly however, the number of RGCs was stable between DIV12 and DIV21, suggesting that the period of RGC cell death is closed at DIV12, which corresponds to P7 *in vivo*. This is earlier than the end of the period of RGC cell death which is estimated at the end of the second postnatal week in mice [3]. In addition, the spatial reorganization of the different cell types within the retinal explant suggests that the laminar organization of the retina is mimicked *ex vivo*, resembling other retinal explants models *in vitro* [38], [39].

### Modulation of developmental reduction of RGC number by electrical activity and target availability

The massive reduction of RGC number *in vivo* is mimicked *ex vivo*. Blockade of electrical activity in the rat retina has previously been found to modulate the survival of RGCs with large scale targeting errors [11], [12]. We show here that blocking electrical activity reduces the decrease in Brn3a-expressing RGCs in mouse retinal explants. These results extend the modulation of RGC population size by electrical activity to a large population of RGCs. However it should be noted that even though activity blockade effect raised the population of Brn3a neurons by 64%, this effect is fairly limited when considering the initial pool of Brn3a neurons (84% cell death in TTX, compared to 90% reduction in control conditions). Thus, most of Brn3a-positive cells are not sensitive to TTX and undergo cell death, suggesting that electrical activity is a modulator of developmental cell death but is not a major molecular pathway for reducing the RGC population size.

Electrical activity is required for competition-driven selection of surviving RGCs. The use of the retino-collicular co-culture allowed the modulation of the target size. We found that increasing the size of available SC increased the surviving RGCs between DIV4 and DIV12, corresponding to P0 and P7 respectively *in vivo*. This observation confirms previous observations indicating that reduction of SC targets decreases RGC survival [7]. Interestingly, late-born RGCs acquire target-dependent survival with a delay compared to RGCs generated earlier [40]. It will be of interest to examine whether this feature is maintained *ex vivo*, to better understand the differences between these two populations of RGCs.

### Regulation of RGC number is competition-dependent but is not influenced by ephrin-A signaling

RGC developmental cell death has been suggested to favor the selective elimination of ectopic projections of retinal axons in the SC [5], [11], [12], [18]. Ephrin-A signaling is critical for the topographic organization of retinal projections both *in vivo* and in the retino-collicular co-culture [22], [33], [41]. Moreover, ephrin-A signaling promotes apoptosis of neuronal progenitors in the cerebral cortex [34]. However, it is still unclear whether topographic defects in retinal projections induced by lack of ephrin-A signaling affect RGC developmental cell death [42]. We tested this hypothesis with the generation of an abnormal retino-collicular projection *ex vivo*, using ephrin-A5 to induce an overall stimulation of ephrin-A/EphA signaling. We found no change in the number of RGCs in ectopically projecting retinal explants. We conclude that the ephrin-A-dependent refinement of retinal projection does not rely on the elimination of ectopically projecting RGCs. This is in agreement with observations showing that the refinement of eye-specific domains in the visual thalamus is unaltered in mice overexpressing Bcl2, an anti-apoptotic gene increasing the number of RGCs when overexpressed [43].

The present *ex vivo* analysis showed that RGC developmental cell death is modulated by electrical activity and target size but is not dependent on ephrin-A signaling. This implies that electrical activity in the retina modulates independently RGC population size and the organization of retinal arbors in visual target fields. The effects of electrical activity on RGC cell numbers may be related to the requirement of trophic factors from the targets since neural activity modulates expression of trophic factors [44]–[46]. On the other hand, the activity-dependent development of retinal maps likely involves different mechanisms, as shown for the regulation of ephrin-As signaling pathway [19], and a synaptic transmission-dependent mechanism to confine projections of individual RGCs in small areas and define ipsi and contralateral domains [47]–[49].

## Materials and Methods

### Animals

Experiments were conducted in compliance with the standard ethical guidelines (European Community guidelines on the care and use of Laboratory animals and French Agriculture and Forestry Ministry guidelines for handling animals-decree 87849, license B75-05-22. Our Institute has the authorization for the use of mice without approval of specific research project). Outbred mice with a Swiss background were obtained from Janvier's animal facility (Orleans, France). β-actin-GFP and Brn3a-LacZ reporter (generous gift of Dr E. Turner) lines were obtained from local colonies. E0.5 is the day following the mating night P0 is the day of birth.

### Histology

Pups were fixed by perfusion through the heart with 4% paraformaldehyde after cold anesthesia at P0, P3, and P7. The eyes were dissected out, post-fixed for 12 hours, and cryoprotected in 10% sucrose. 15 µm thick serial sections were obtained from the frozen eyes using a cryostat. Sections were washed 1 h in PBS-0.2% gelatin and 0.25% Triton X-100, and incubated overnight at room temperature in anti-Brn3a (1/200; Abcys), anti-recoverin (1/1000; Abcys), or anti-ChAT (1/200; Chemicon-Millipore). They were washed again and incubated for 2 h at room temperature in the appropriate secondary antibody coupled to Alexa 488 (1/200; Invitrogen) or Cy3 (1/200; Jackson). Sections were finally washed in PBS and mounted in mowiol-Dabco (25 mg/ml; Sigma).

### Organotypic co-cultures

Organotypic retino-collicular co-cultures were prepared as previously described [22]. Briefly, a block containing the superior and inferior colliculus was dissected out of brains from P6 pups in cold PBS-0.5% glucose, and sliced into 300 µm-thick para-sagittal slices using a tissue chopper. Slices selected from the center of each SC were transferred on a Millicell culture insert with 0.4 µm pore size (Millipore), and maintained in 5% CO2 at 35°C. E15.5 embryos were obtained from pregnant OF1, β-actin-GFP or Brn3a-LacZ reporter mice. The retinas were dissected out, placed in culture medium and cut into 200 µm^2^ explants. Individual retinal explants were placed rostral and in contact with one, two or four SC slices, that had been cultured the day before. In the presence of more than one SC slice per retinal explant, all the slices were oriented in order to have the retinal explant placed along the rostral part of the dorsal SC. The co-culture preparations were then cultured for 4, 7, 12 or 21 days at 35°C in 5% CO2. The culture medium used was composed of 60% basal medium eagle (BME) (Invitrogen), 20% inactivated horse serum (Invitrogen), 20% 1× HBSS (Invitrogen), 0.4% glucose (Invitrogen,), 0.8 mM glutamine (Invitrogen) and was replaced every 2 days. For pharmacological manipulations, TTX (2.5 µM; Euromedex) or ephrin-A5 (0.5 µg/ml; R&D Systems) were added the day after the beginning of the culture (DIV1) and at each medium change.

Cultures were fixed for 10 minutes in 4% paraformaldehyde, with 15% sucrose at room temperature, washed 10 minutes in PBS before non specific binding was blocked with 10% fetal bovine serum in PBS, 0.1% Tween 20 (1 h) at room temperature. Primary anti-Brn3a (1/200; Absys), anti-recoverin (1/1000; Abcys), anti-ChAT (1/200; Chemicon-Millipore), or anti-GFP (1/1000; Invitrogen) antibody was diluted in the blocking solution and applied for 1 h at room temperature. The cultures were washed 10 minutes in PBS-0.1%Tween 20 and secondary antibody (goat anti-mouse coupled to Alexa 488 fluorochrome (1/200; Invitrogen) was diluted in the blocking solution and added for 1 hour at room temperature, before counterstaining with bisbenzimide (10 µg/ml; Sigma). The co-culture preparations were finally washed in PBS and mounted in mowiol-Dabco (25 mg/ml; Sigma).

For β-galactosidase revelation, cultures were fixed for 10 minutes in 4% paraformaldehyde, rinsed 3 times in PBS and incubated overnight in the staining solution (5 mM K_4_Fe(CN)_6_, 5 mM K_3_Fe(CN)_6_,2 mM MgCl2, 1 mg/ml X-gal in PBS). The tissue was rinsed 3 times in PBS and mounted in mowiol.

### Electrophysiological recordings

Extracellular recordings of the mesencephalic slice were done as previously described [19]. Briefly, cultures were placed in an interface recording chamber and superfused with an artificial cerebrospinal fluid (ACSF) containing 125 mM NaCl, 4.5 mM KCl, 2.5 mM NaHCO_3_, 1 mM CaCl_2_, 1 mM MgCl_2_ and 10 mM D-glucose, equilibrated with 5% CO_2_ in O_2_. Records were made using tungsten electrodes. Data was acquired using Axoscope software (Molecular Devices) and digitized at a temporal resolution of 0.05 ms.

### Data Analysis

#### Count of Brn3a-, recoverin- and ChAT-positive cells in the retina

The 2 sections of the retina with the largest diameter were identified and micrographs were obtained using a wide field fluorescence microscope and a 5× objective. Their dimensions were used to compute the total surface of the retina. The retina was approximated to a spherical cap and its area was computed using the following formula: S = 2πrh, where r is the radius of the sphere, and h the height of the spherical cap. Micrographs of the immunostained retinas were obtained using a wide field fluorescence microscope and a 20× objective. The density of Brn3a- and ChAT- positive cells (number of cells per µm^2^ of retinal surface) was measured from ≥4 fields of the retina for each animal using the ITCN plugin of ImageJ (W. Rasband, NIH) for Brn3a-positive RGCs or manually for ChAT-positive amacrine cells, and multiplied by the computed surface to obtain an estimate of the total number of each cell type in the retina. Each recoverin-stained retinal section was manually thresholded and the area covered by immunolabeling was measured using ImageJ. The measured area was divided by the length of the retina (to measure the area/unit length) and multiplied by the computed surface of the retina to estimate the volume occupied by recoverin-positive cells in the entire retina. Statistical comparisons were performed using the Kruskall-Wallis test.

#### Density of Brn3a-, ChAT- and recoverin-positive cells and pyknotic profiles in retinal explants *in vitro*

Micrographs of the retinal explants were obtained using a wide field fluorescence microscope. For each explant Brn3a-, ChAT-positive cells or pyknotic profiles were manually counted in two 310×245 µm fields for ≥10 explants per conditions. One region was chosen as the region with the highest Brn3a-expressing cells density in the peripheral zone of the explant. The other field was the densest region in the center of the explant. The count of ChAT-positve cells was performed in two fields in the peripheral zone of the explants. Densities of Brn3a- and ChAT-positive RGCs were normalized to those obtained in parallel control cultures (DIV4 for time course experiments, free of drugs for pharmacology experiments). Densities of pyknotic profiles were normalized to the density of Brn3a-positive cells at DIV4 to give an approximation of the percentage of RGCs lost at each age. The density of recoverin staining in the retinal explant was measured after thresholding the fluorescence of the micrographs and normalized to the value obtained in parallel cultures at DIV4. Statistical comparisons were performed using ANOVA.
